# Sexual dysfunctions in MS in relation to neuropsychiatric aspects and its psychological treatment: A scoping review

**DOI:** 10.1371/journal.pone.0193381

**Published:** 2018-02-27

**Authors:** Jana Pöttgen, Anita Rose, Wim van de Vis, Jannie Engelbrecht, Michelle Pirard, Stefanie Lau, Christoph Heesen, Sascha Köpke, RiMS Special Interest Group Psychology and Neuropsychology

**Affiliations:** 1 Institut für Neuroimmunologie und Multiple Sklerose, Universitätsklinikum Hamburg-Eppendorf, Hamburg, Germany; 2 Klinik und Poliklinik für Neurologie, Universitätsklinikum Hamburg-Eppendorf, Hamburg, Germany; 3 The Raphael Medical Centre, Tonbridge, United Kingdom; 4 Revalidatie Centre Roessingh, Enschede, Netherlands; 5 Sclerose Hospital Erne, Ry, Denmark; 6 National MS Centre, Melsbroek, Belgium; 7 University of Lübeck, Nursing Research Unit, Institute for Social Medicine and Epidemiology, Lübeck, Germany; Charite Medical University Berlin, GERMANY

## Abstract

**Objective:**

Sexual dysfunction in multiple sclerosis (MS) is a significant, but often underestimated and overlooked suffering. Interventions to treat sexual dysfunction in MS are rare. The relation between sexual dysfunction in MS and psychological as well as neuropsychological aspects is evident. However, this field of research remains markedly underdeveloped in this severe chronic illness. The aim of this scoping review is to describe the relevant knowledge in this area and to identify psychological interventions to treat sexual dysfunctions in MS.

**Methods:**

A scoping review was conducted to answer the following questions: (1) Which psychological and neuropsychological factors impact on sexual dysfunction in MS and vice versa? (2) What kind of psychological interventions aiming to improve sexual dysfunctions in MS are available? A comprehensive search and review of MEDLINE, PsycINFO, and CINAHL was completed by using a recent methodological framework for scoping reviews.

**Results:**

23 publications covering a total of 13,259 people with MS and 532 healthy controls were identified. Sexual dysfunction was found to be very common in MS and there is an obvious relation to psychological disorders as e.g. depression and anxiety and also to psychological aspects as partner relationship and quality of life. The relation between sexual dysfunction in MS and neuropsychological impairment has only rarely been studied and no clear results were found. Only two studies were identified, assessing the effectiveness of psychological intervention studies on sexual dysfunction in people with MS, and a third study presenting a secondary analysis of a study targeting depression. All three studies reported significant improvements in sexual dysfunction as well as partly in psychological variables.

**Conclusions:**

There is a pressing need for the development and adequate evaluation of psychological interventions for sexual dysfunctions in MS. In addition, sexual dysfunction and its impact on psychological wellbeing should be more focussed in clinical care.

**Registration:**

This review is registered with PROSPERO; Registration number: CRD42016033066.

## Introduction

Multiple sclerosis (MS) is a heterogeneous inflammatory and degenerative disease of the central nervous system (CNS) and affects mostly young adults. MS is characterized by demyelination and damage of axons and affects physical as well as psychological and cognitive aspects of patients’ lives [1,2]. Both depression and cognitive impairment are common burdens in people with MS. Sexual dysfunction (SD) in MS is one of the more hidden symptoms, which are often overlooked in clinical examinations, but have a large impact on patients’ well-being [3].

### Human sexuality, sexual dysfunction and therapy

Human sexuality is complex and consists of anatomical, physiological, psychological, developmental, cultural, and relational factors [4]. Seven components of human sexuality have been identified: gender identity, orientation, intention, desire, arousal, orgasm, emotional satisfaction (DSM-IV). To describe human sexual behaviour, Masters & Johnson [5] developed the sexual response cycle which consists of four phases (excitement, plateau, orgasm, and resolution) and was later adapted to desire, arousal, orgasm, and resolution including sub-components [6]. Phase 1 (desire) reflects the biological, psychological, and social aspects of desire. Phase 2, arousal, is stimulated by psychological and/or physiological components and prepares for phase 3, i.e. orgasm. In this phase physiological factors related to phase 2 continue, while in men, ejaculation is perpetuated and in women, the uterus and lower third of the vagina contract involuntarily. The final phase 4, resolution, is highly dependent on the achievement of orgasm. If orgasm is achieved, physiological changes return to baseline, vasocongestion diminishes and calmness and relaxation set in. If orgasm is not achieved, irritability and discomfort may result. SD can occur in each of the phases and can be related to each of the mentioned functions. The DSM V defines general SD as an impairment of the ability for sexual reaction and beneficial sexual experiences of a person [7]. A diagnosis of SD requires a person to feel extreme distress and interpersonal strain for a minimum of 6 months (excluding substance or medication-induced SD). While the focus of these criteria is on the diagnosis of SD, it should be kept in mind that the expression of a person’s sexuality is intimately related to his or her partner’s sexuality [8]. In addition, the impact of psychological as well as cognitive factors as depression, anxiety, stress etc. on SD should also be considered during the treatment of SD [9]. For the treatment of SD, psychotherapeutic interventions as e.g. cognitive behavioural therapy have been proposed as gold standard [10], but also pharmacotherapy as e.g. testosterone, androgen or estrogen supplementation or the modulation of dopaminergic pathways to treat erectile dysfunction can be appropriate treatment options for specific problems [11,12].

### Sexual dysfunction in MS

SD in MS is a significant but often underestimated and overlooked suffering. SD is experienced by 50 to 90% of men with MS and 40 to 80% of women with MS[13] which is significantly more than in general population samples[14,15]. Impotence and erectile dysfunction have been consistently reported as the main SD among men with MS. Other problems include decreased libido, fatigue and ejaculatory dysfunction[16–18]. Among women with MS, problems tend to be more wide ranging and include decreased libido, decreased lubrication, orgasmic dysfunction, decreased genital sensation or dysesthesia, dyspareunia and vaginismus[16,18]. Different causes for SD and related problems have been described. DasGupta & Fowler[19] reported disease complications like urinary and bowel symptoms as affecting factor for SD. But, also complaints as sensory dysfunction, psychological problems, side effects of medications, and other disease complications as e.g. spasticity have to be considered as possible causes of SD in MS[20,21]. Also the state and imbalance of hormones in MS (e.g. ^17^beta estradiol, testosterone, progesterone, and prolactin) may impact SD in MS and could affect sexual well-being and cause sexual problems[14]. However, the aetiology of SD in MS is more complex and is also related to anatomic, physiologic, biologic and psychological factors. The variability and characteristic of SD also appears to be affected by MS-related disability levels and disease duration[22]. Foley & Iverson[23] developed a comprehensive conceptual model of SD in MS consisting primary, secondary and tertiary SD. The model delineates MS-related neurological changes in the CNS (primary SD), MS symptoms that are not related to the neural pathways of the genital system but to MS-related physical changes (secondary SD), and disability-related psychological, emotional, social and cultural influences (tertiary SD). SD is considered to have a great impact on quality of life, cause distress and affect human relationships[22]. In addition, SD is related to psychological impairment as depression or anxiety. A relation between SD and neuropsychological impairment seems to exist, but research regarding causes and effects of SD in relation to neuropsychiatric impairment in MS is rare.

### Treatment of SD in MS

Despite their chronic illness and disabilities, people with MS are still persons with a sexual life with the ability to share love, bonding, intimacy and sexual experiences. Many patients will not raise the issue of sexual problems unless specifically asked by their health care provider and many physicians are reluctant to discuss this aspect of impairment[24,25]. Counselling and support interventions have been shown to positively influence sexuality and sexual satisfaction[13,26,27]. In addition, the use of sexual gadgets as e.g. vacuum erection devices has been studied[28]. A number of pharmacological treatments have been proposed especially for men with MS and SD as e.g. Sildenafil for the treatment of erectile dysfunction and dopamine agonists (apomorphine)[29]. Women with MS are faced with limited treatment options including sildafenil[30] and possibly estrogen replacement therapy[31,32], although more research is needed.

It is obvious that there is a strong relation between psychological and neuropsychological factors and sexuality in MS, although their impact and the effects of psychological interventions addressing SD in MS have rarely been studies. Therefore, we aimed to systematically review the available evidence on SD in MS.

## Methods

We conducted a scoping review on the impact of psychological and neuropsychological factors on SD in people with MS following the frameworks of Arksey & O’Malley[33] and Levac[34] as outlined below. Considering the absence of systematic reviews on this topic, this approach seemed most appropriate. All titles retrieved by the search were screened by two independent reviewers and discrepancies discussed afterwards. We used the Critical Appraisal Skills Programme (CASP) tools appropriate for the different study design to determine validity and quality of the studies [35]. We slightly adapted the CASP tools regarding our research question to enhance the validity and reliability of the quality assessment. We used the CASP trial checklist for the intervention studies. Cohort studies including longitudinal studies, cross-sectional studies and studies without control groups were assessed using the CASP Cohort Study Checklist. For cross-sectional studies with control groups we used the CASP Case Control Study Checklist. Scores ranged from 0-to 7 points with higher values indicating better quality. Two reviewers (J.P., S.K.) assessed studies separately accordingly to the CASP tools requirements and discussed discrepancies of assessments afterwards.

The protocol for this review has been registered with PROSPERO (CRD42016033066).

### Eligibility

A scoping review can be used to examine the extent, range and nature of research activity, determine the value of undertaking a full systematic review, summarize and disseminate research findings, or identify gaps in the existing literature. Scoping reviews differ from systematic reviews as authors do not typically asses the quality of the evidence in included studies. Main aims of scoping reviews address the identification of broader topics and research questions with the purpose of identifying research gaps and making recommendation for future research[34].

Following the original approach described by Arksey & O’Malley[33], Levac et al.[34] suggested 6 stages of a scoping review: (1) identifying the research question and defining the search strategy; (2) identifying relevant studies; (3) study selection; (4) charting the data; (5) collating, summarizing, and reporting the results (e.g., comparing across interventions); and (6) consultation (optional). The application of the different steps is described in the following.

#### Step 1: Identifying the research question

This review aimed to gather more knowledge about psychological and neuropsychological impact on SD in people with MS. The main questions were: (1) what are typical psychological and neuropsychological factors related to SD in people with MS, and (2) are there psychological interventions for SD in people with MS?

#### Step 2: Identify relevant studies

We searched: MEDLINE, CINAHL and PsychInfo including the following terms: multiple sclerosis, sex, sexual disorders, sexual symptoms, lubrication, orgasm, sexual satisfaction. Studies were limited to those in English, German, Spanish, French, Dutch, or Italian and to publications since 1985. In addition, we screened the reference lists of included papers and performed a hand searched for recently published articles in relevant journals. The last search was performed on the 7^th^ of November 2016.

#### Step 3: Study selection (including / excluding)

***Retrieval Procedure*.** In a first step, three reviewers (W.v.d.V., C.C., J.P.) scanned the titles and abstracts independently to determine whether the study evaluated SD in MS in relation to psychological or neuropsychological problems. This sensitive procedure was chosen to include a wide range of articles with different aspects concerning SD in MS in relation to psychological or neuropsychological problems (e.g., quality of partnership and sexuality, SD in relation to quality of life, different therapeutic approaches to treat SD in MS). The main inclusion criterion was the relation of SD in MS to psychological or neuropsychological problems. All next steps were discussed and finalized in meetings with the whole research group. For the abstract and full text screening, studies also had to: 1. include patients and report clear eligibility criteria, 2. present quantitative data, 3. focus on neuro/psychological aspects or interventions addressing sexuality, and 4. include neuropsychological measures obtained by neuropsychological testing. We defined SD very broadly so that all aspects of problems with sexuality would be considered for the review.

**Fig 1:**
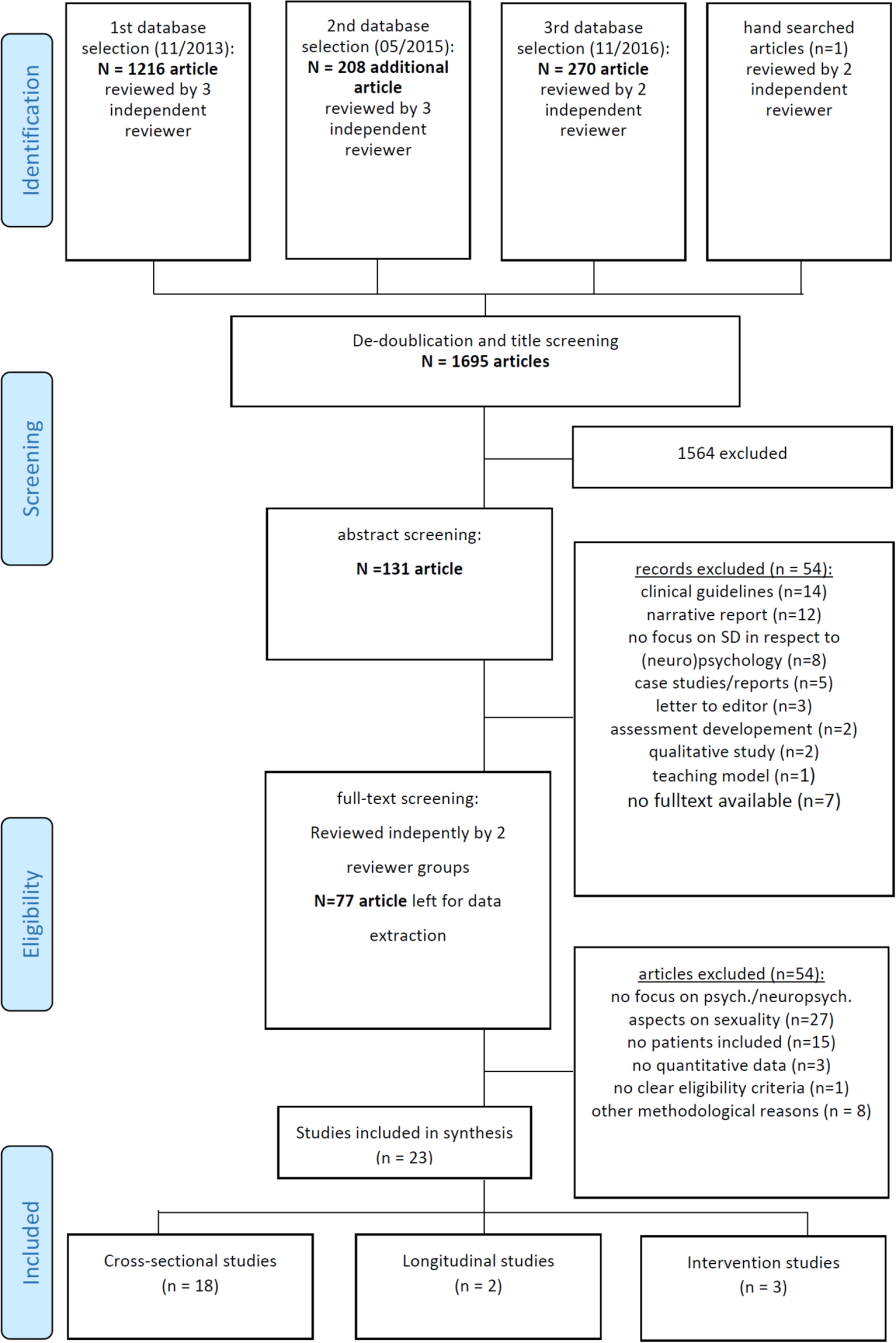
Data selection flow.

The first database search generated 1695 references after de-duplication, of which 131 records remained of initial title screening. Of these, 39 articles had been selected by all authors with an overlap of 92 articles that were not selected by at least one reviewer. Discrepancies were discussed each among authors and finally another 54 articles were excluded for different reasons (see Fig 1). In summary 77 articles were assessed as full text and reviewed by two independent reviewers which led to exclusion of another 54 articles. Finally, 23 articles were included for the scoping review (Fig 1).

#### Step 4: Charting the data

***Data-extraction***. Characteristics of the included articles were extracted using an own data extraction sheet assessing 1. Article name and aim of the study, 2. Study type, 3. Recruitment procedure, 4. Time and location of study, 5. Setting and data collection method, 6. Intervention and treatment duration (if applicable), 7. Ethical approval, 8. Definition of SD, 9. Statistical methods, 10. Inclusion and exclusion criteria, 11. Sample characteristics, 12. Outcome measures (SD, psychological and neuropsychological variables) and the results. Data extraction from the articles was executed in groups of two independent authors each. Results were presented in the whole group and finally cross-checked by the lead author.

## Results

### Step 5: Collating, summarizing, and reporting results

#### Characteristics of the studies

All included studies (n = 23) were published in English. Demographic and disease specific data are summarized in Table 1. The studies included a total of 13,259 people with MS and 532 healthy controls. Patients’ disability levels were reported in only 12 of 23 studies using different measures (EDSS, UNDS, PDDS etc.).

**Table 1:** Demographic and disease specific data of people with MS (n = 23 studies).

Measure	n (studies)	Range
Age (mean years)	22	32.8–50.8
Sex ratio f/m (%)	20	55.6–100
Years of Education (mean)	4	13.0–15.8
Education (%)		
---primary/secondary degree	7	29.8–82.4
---university degree		17.6–70.2
Employed (%)	7	17.1–72.8
Family status (%)		
---married or in relation	8	66.7–100
MS Course (%)	8	
---RRMS		63.5–95.0
---SPMS		5.0–26.6
---PPMS		0–23.2
Disease duration (mean years)	15	1.8–12.8
EDSS (median)*	11	1.0 to 5.6

*in 2 studies ranges were given (e.g. EDSS 0–4.5 & 5.0–8.0, EDSS < 4.5 & >4.0–9.0)

Studies typically reported disease duration. Only eight studies reported the MS course.

Specific study characteristics are displayed in Table 2. Most of the 23 studies were conducted in the US (n = 5), Iran (n = 4), or Italy and Australia (n = 3 each). In six studies healthy controls were examined for comparison. The distribution of SD was between 17 and 100%, while in n = 6 studies SD was not quantified. Five studies only included women and in 17 studies more women than men were examined (female rate 56 to 88%). All of the studies used specific sexual dysfunctions scales or structured interviews. Inclusion and exclusion criteria were reported for 18 studies, more or less specifically.

**Table 2:** Overview of included studies.

Reference	Country	Setting, recruitment	Screened or contacted / included	Study aim	CASP total score	Definition of sexual dysfunction
Barak et al. [36]	Israel	cross-sectional, recruitment not reported	nr / 41 (53% with SD; 74% female; n = 0 HC)	to evaluate the frequency and character of sexual dysfunctions in an early stage of relapsing-remitting (RR) multiple sclerosis and to correlate sexual disturbances with various disease parameters	1/7	self-report questionnaire, two items for 5 parameters (loss of libido, arousal difficulties, impotence, premature ejaculation, anorgasmia), when both items were endorsed = SD.
Blackmore et al. [26]	USA	intervention, recruited through Kaiser Permanente Medical Care Group the National Multiple Sclerosis Society (NMSS).	127 / 81 (73% with SD, 77% female, n = 0 HC)	to investigate whether both negative and positive partner support predict sexual satisfaction in individuals with MS, who participated in a larger randomized psychotherapy study designed to treat depression in MS	6/7	Sexual abilities section of the GNDS—the item “Do you have any problems in relation to your sexual function?” (yes/no)
Dupont, [37]	UK	cross-sectional, elected from neurology department files of three hospitals	199 / 116 (SD nr, 62% female, n = 0 HC)	to determine what sexual difficulties are experienced by male and female patients with MS, how common are relationship difficulties among men and women where one, what coping strategies are used by people with MS to deal with the sexual, what sexual relationship factors are associated with MS disease characteristics	5/7	GRISS & GRIMS
Foley et al. [13]	USA	intervention, recruited from a hospital-based comprehensive care MS Center	11 / 9 (SD nr, sex nr, n = 9 HC)	to test the efficacy of a psychoeducational and counselling intervention to rehabilitate sexual dysfunction, marital satisfaction, and marital communication in people with MS and their sexual partners	2/7	self reported by patients
Fragala et al. [38]	Italy	cross-sectional, consecutive patients with MS in remission phase	nr / 135 (76% with SD, 56% female; n = 0 HC)	to determine the relationship between SD, neurological disability, anxiety, and depression in a cohort of people with MS affected by lower urinary tract dysfunction and to investigate potentially predictive factors of SD.	7/7	IIEF-15 < 60; FSFI < 26.55
Ghajarzadeh et al. [39]	Iran	cross-sectional, recruitment not reported	nr / 100 (66% with SD; 100% female; n = 50 HC)	to determine sexual function Index of Iranian people with MS.	5/7	FSFI < 26.55
Gumus et al. [40]	Turkey	cross-sectional, recruitment not reported	nr / 70 (SD not reported; 100% female; n = 72 HC)	to determine effects of MS on female sexuality and to compare the results with those of healthy women.	4/7	not defined
Khakbazan et al. [27]	Iran	Intervention, recruited in Iranian Community of Support for MS Patients	nr/90 (100% with SD; 100% female; n = 0 HC)	was carried out to evaluate the effectiveness of the PLISSIT model as a valuable framework for health professionals, especially midwives to address the sexual problems of the women who are sexually active and suffer from MS	4/7	FSFI, cut-off ≤28 / Subscales: cut-off points for the subscales: Desire = 3.3, Arousal = 3.4, Lubrication = 3.7, Pain = 3.8, Orgasm = 3.4, Satisfaction = 3.8.
Kisic-Tepavcevic et al. [41]	Serbia	Longitudinal based on Tepavcevic et al. (2008)	215/93 (88% with SD; 71% female, n = 0 HC)	to investigate changes in the level of sexual functioning after a period of the 3- and 6-year follow-up; symptoms of SD which changed over time; and demographic and clinical characteristics of people with MS as potential predictors of changes in SD	7/7	SSFS
Kolzet et al. [42]	USA	cross-sectional, mailing to patients who are registered in the NARCOMS registry	4267 / 4267 (SD nr; 75% female; n = 0 HC)	to evaluate predictors of body image related SD, including sociodemographic, mental health, help-seeking behaviors for sexual problems, time since diagnosis, and self-reported disease status in a large national sample of men and women with MS	5/7	not defined
Lew-Starowicz et al. [43]	Poland	cross-sectional, subsequently recruited from the National Multiple Sclerosis Center	nr / 204 (ca. 68% with SD, 67% female, n = 0 HC)	to investigate correlates of sexual functioning (SF) in people with MS with special focus on specific neurologic deficits, depressive symptoms, and relationship factors; to investigate their impact on SQoL; and to search for possible gender differences.	5/7	not defined
McCabe et al. [44]	Australia	cross-sectional, randomly selected from people with MS who were registered with the MS Society.	117 / 111 (ca. 73% with SD 70% female; n = 0 HC)	to assess the perceived impact of MS on sexual functioning and on social and intimate relationships. The impact of these factors on quality of life was also evaluated.	5/7	self diagnosed by self-made questionnaire (regarding typical sexual disturbances)
McCabe [45]	Australia	cross-sectional, patients who are registered with the MS Society and HC randomly selected	630 / 381 (ca. 83% with SD; 62% female; n = 291 HCs)	to determine differences between people with MS and people from the general population in the nature and quality of their relationships, in their sexual functioning and sexual satisfaction. Gender differences and the contribution of coping style were also evaluated.	4/7	SDS (SD exist when patients report problems in the subscales)
Mc. Cabe et al. [46]	Australia	longitudinal over 6 month, patients who are registered with the MS Society of Victoria and HC randomly selected	630 / 321 (ca. 82% with SD; 63% female; n = 239 HCs)	to determine how the use of particular coping strategies at one point in time impacts on sexual and relationship variables at a later point in time investigated the interrelationships among a number of sexual and relationship variables (sexual satisfaction, sexual activity, sexual dysfunction, relationship satisfaction) and how these variables predict one another over time and to compare results with the data from the general population	4/7	SDS (SD exist when patients report problems in the subscales)
Mohammadi et al. [47]	Iran	cross-sectional, consecutive recruitment in the outpatient department	320 / 226 (55% with SD, 100% female, n = 0 HC)	to determinant disease-related and psychological risk factors for sexual dysfunction in women with MS, the extent of the problem and provide appropriate guidelines for planning managed care	6/7	FSFI, cut-off ≤28 / Subscales: cut-off points: Desire = 3.3, Arousal = 3.4, Lubrication = 3.7, Pain = 3.8, Orgasm = 3.4, Satisfaction = 3.8.
Quaderi et al. [48]	Iran	cross-sectional, patients from the Iranian MS Society were asked to participate	145 / 132 (83% with SD; 100% female; n = 0 HC)	to examine the relationships between three levels of female sexual problems and all subscales of quality of life among Iranian women referred to the Iranian MS Society and to answer the question; ‘‘how sexual problems relate to quality of life in women with MS"	5/7	MSISQ
Schairer et al. [3]	USA	cross-sectional, recruited via internet and survey from a large MS patient registry	9201 / 6183 (SD nr; 75% female; n = 0 HC)	to exam the impact of sexual dysfunction on HrQoL in a large United States (US) national sample using a validated sexual dysfunction measure that is specific to MS	5/7	not defined
Stepleman et al. [49]	USA	cross-sectional, patients at the Regional MS center were invited to participate	73 / 73 (SD nr, 88% female, n = 0 HC)	to examine sexual health, health care communication, and MS-related variables within the context of sexual health care communication and overall sexual satisfaction in persons with MS	4/7	MSISQ
Tepavcevic et al. [50]	Serbia	cross-sectional, consecutive unselected patients with MS	215 / 109 (85% with SD, 72% female, n = 0 HC)	to estimate the type, intensity, frequency of SD in people with MS and to investigate its influence on all the domains of QoL measured by MSQoL-54 and to analyze relationships between sexual functioning and patients’ demographic and clinical characteristics, neurological status, fatigue, psychological and cognitive functioning	5/7	SSFS
Vitkova et al. [51]	Slovakia	cross-sectional, selected from the clinical MS database	223/223 (17% with SD; 67% female; n = 0 HC)	to explore the association of bladder, bowel and sexual dysfunction with the physical and mental dimension of HRQoL in patients with MS stratified by disease duration (5 and 45 years) and controlled for clinical and sociodemographic variables	6/7	ISS score = 2 or 3
Young et al. [52]	UK	cross-sectional, were asked in several UK MS centres to take part	722 / 538 (82% with SD, 72,5% female, n = 0 HCs	to ascertain the relationships between sexual function and fatigue, physical disability and depression, examining how these are influenced by demographic factors such as gender and age, together with subtype of MS.	6/7	MSISQ
Zivadinov et al. [53]	Italy	cross-sectional, consecutive patients	300 / 108 (72,5% with SD, 65% female, n = 110 HCs)	to examine the relationships of SD with sphincter dysfunction, neurological status, disease and patient’s characteristics, psychological and cognitive functioning.	5/7	self-made questionnaire for SD (SD = one or more symptoms of SD
Zorzon et al. [54]	Italy	cross-sectional, consecutive patients	nr / 62 (66 % with SD; 65 % female; n=0 HC)	to examine the relationship of SD with the severity and location of the pathological lesions shown by magnetic resonance imaging (MRI) of the brain and a series of clinical variables in MS patients.	4/7	SSFS

#### Research design and outcomes

Research designs are displayed in Table 2. Outcomes for sexual functions and for psychological and neuropsychological assessments used in included studies are provided in the appendix.

Only three of the 23 studies investigated interventions to improve sexual function. Three cross-sectional studies evaluated sexual function in relation to neuropsychological functioning, whereas the other 15 focused on the relation of SD and psychological measures. One of the two longitudinal studies mainly focused on coping and its impact on sexual functioning over time, while the other investigate changes in the level of sexual functioning after 3 and 6 years; changes in symptoms of SD over time; and related demographic and clinical characteristics.

The most commonly used questionnaires to assess sexual functioning were the Multiple Sclerosis Intimacy and Sexuality Questionnaire (MSISQ) (n = 6) and the Female Sexual Function Index (FSFI) (n = 4). Three studies used own questionnaires/interviews.

The main foci of psychological assessment were quality of life (most frequently measured by MSQLI (n = 2) and SF-12 (n = 2)) and depression (most frequently measured by BDI (n = 7) and HDRS (n = 3)). Also coping, fatigue, relationship and social support were focused in relation to sexual functioning, in addition demographic variables as marital status. Neuropsychological aspects were only measured in two studies.

#### Results of cross-sectional studies

As shown in Table 3, most cross-sectional studies found significant positive relations between SD and psychological and neuropsychological measures.

**Table 3:** Relations between SD and psychological and neuropsychological measures.

Calculated relations regarding SD and psychology	rating
***relation between Sexual dysfunction and depression***	
Mohammadi et al. 2013 [47]	+ / +++
Zorzon et al. 2003 [54]	+++
Zivadinov et al. 1999 [53]	++
Lew-Starowicz et al. 2014 [43]	+ to ++
Tepavcevic et al. 2008 [50]	+++
Ghajarzadeh et al. 2013 [39]	+ to +++
Barak et al. 1996 [36]	+++
Fragala et al. 2014 [38]	+ to ++
Gumus et al. 2014 [40]	+++
Dupont 1996 [37]	0
Young et al. 2016 [52]	0
***relation between Sexual dysfunction and anxiety***	
Zorzon et al. 2003 [54]	++
Zivadinov et al. 1999 [53]	++
Tepavcevic et al. 2008 [50]	++
Fragala et al. 2014 [38]	++
Barak et al. 1996 [36]	0
***relation between SD and coping***	
McCabe 2002 [45]	- to --/++
***relation between sexual dysfunction and fatigue***	
Zivadinov et al. 1999 [53]	+
Tepavcevic et al. 2008 [50]	+ to ++
Gumus et al. 2014 [40]	0
Young et al. 2016 [52]	0
***relation between sexual dysfunction and cognitive performance***	
Zivadinov et al. 1999 [53]	-
Tepavcevic et al. 2008 [50]	--
Dupont 1996 [37]	0
***relation between sexual dysfunction and quality of life***	
Lew-Starowicz et al. 2014 [43]	- to ---
Tepavcevic et al. 2008 [50]	- to --
Schairer et al. 2014 [3]	--
Quaderi et al. 2013 [48]	--
Vitkova et al. 2014 [56]	--
Kolzet et al. 2015 [42]	--
***relation between sexual dysfunction and relationship***	
McCabe et al. 1996 [44]	+++/--
McCabe 2002 [45]	+++/--

(+ = positive correlation, - negative correlation, +++/--- = p < .001, ++/-- = p between .001 and .01, +/- = p between .011 and .05, 0 = p>.05

*SD and depression*: In 9 studies depression was found as a significant factor contributing SD in MS.

In three studies only women with MS were studied and significant positive relations between SD measured by FSFI and depression were found [39,40,47]. Six other studies assessed both women and men with MS and found also significant positive relations between different SD subscales (e.g. erectile function, desire, orgasm) and depression [36,38,43,50,53,54].

Dupont [37] could not show a clear relation between SD and depression in 116 people with MS who lived in a partnership. Young et al. [52] investigated SD in relation to depression, fatigue and physical function in 538 people with MS and found no direct association between depression and sexual functioning, but depression appeared as a consequence of the psychological issues associated with SD.

*SD and anxiety*: The relation between SD and anxiety was assessed in five of the studies with significant positive correlations between SD and anxiety observed in four studies. One could only show a relation between SD and anxiety for women with MS [53], while the other three studies found positive relations between SD and anxiety in both women and men [38,50,54]. On the contrary, Barak et al. [36]did not find any significant correlation between SD and anxiety.

*SD and coping*: Coping in relation to SD was investigated in two studies. McCabe [45] found a significant negative correlation between SD and one subscale of the WOCQ (focusing on the positive) in men with MS and significant negative correlations between SD and two subscales of the WOCQ (focusing on the positive and cognitive functioning) and a significant positive correlation between SD and problem focused coping in MS women.

*SD and fatigue*: Two studies found a significant positive relationship between SD and fatigue. One found significant impact only in women with MS [53] whereas Tepavcevic et al. [50] found the relation in both women and men. Gumus et al. [40] also investigated the relation between SD and fatigue in 70 women with MS and 72 healthy controls, but failed to identify a significant relation. Also Young et al. [55] found no impact of fatigue on SD in MS.

*SD and cognitive performance*: The impact of objective cognitive performance on SD was investigated in three studies. Two studies found significant negative correlations between cognitive performance and SD in MS, but only in women [50,53]. In contrast, Dupont [37] found no significant correlation between cognitive performance and SD in MS.

*SD and quality of life*: Six studies investigated the relation between SD and quality of life in MS and all of them found significant relationships [3,42,43,48,50,56]. Lew-Starowicz [43] found significant negative correlations between SD and sexuality related QoL domains. Two of the studies [48,50] used a MS specific QoL measure and found significant negative correlations between most of the MSQoL subscales (e.g. physical health, physical role limitations, social function, cognitive function) and SD. Schairer et al. [3] and Vitkova et al. [56] found significant negative correlations between SD and both mental and physical QoL domains measured by SF-12/36. Kolzet et al. [42] only investigated the mental health subscale of the SF-12 and found significant negative correlations with SD in MS.*SD and relationship*: Three studies assessed SD and marital or couple relationship and found significant correlations. McCabe et al. [44] investigated the impact of SD in MS on social and intimate relationship, and found positive correlations between the frequency of sexual intercourses and the feel about relationship and negative correlations between frequency of sexual intercourses and partner concerns about sex. Another study by McCabe [45] found significant positive correlation between relationship satisfaction and sexual satisfaction and negative correlation between relationship satisfaction and sexual difficulties.

*Sexual functioning and physician-patient-communication (not reported in* Table 3*)*: Stepleman et al. [49] investigated factors associated with patients’ sex-related communications with their MS physicians and to overall patient sexual satisfaction. In a sample of n = 73 people with MS they found that more than half of people with MS reported SD, but only a third of patients addressed their sexual concerns with their physician. The frequency of communication about sexual concerns showed associations with satisfaction with physician variables, whereas self-efficacy for these interactions was associated with emotional health variables.

#### Results of longitudinal studies

McCabe et al. [46] investigated how the use of particular coping strategies at one point in time impacts on sexual and relationship variables after up to 6 months later. The second research question focused on the interrelationships among a number of sexual and relationship variables (sexual satisfaction, sexual activity, sexual dysfunction, and relationship satisfaction) and how these variables predict one another over time. 321 people with MS and 239 people from the general population participated in the study. 265 people with MS had at least one sexual dysfunction. The study found that sexual activity at baseline contributed significantly to relationship satisfaction after six month for men with MS. For people diagnosed with MS for less than 7 years, levels of sexual activity after six month were predicted by levels of sexual and relationship satisfaction, as well as levels of sexual activity at baseline. Results also showed that strategies used to cope with illness have no major role in sexual and relationship satisfaction.

The purpose of the study by Kisic-Tepavcevic et al. [41] was to explore longitudinal changes in the level of sexual functioning after a period of 3 and 6 years of follow-up, and to investigate predictors of changes in SD. The study population comprise a cohort of 93 people with MS who were assessed at baseline, and at the 3- and 6-year follow-up. The number of reported SD symptoms increased markedly for both genders during the whole observation period of six years. Duration of follow-up, age, level of physical disability, depression, and fatigue were identified as independent prognostic factors for deterioration of sexual functioning in people with MS.

#### Results of intervention studies

A summary of the three intervention studies is provided in Table 4. In a pilot study, Foley et al. [57] examined the efficacy of a counselling intervention in nine people with MS and their partners in a quasi-experimental research design. The intervention included 12 counselling sessions focused on education, management and cognitive behavioural therapy and consisted of contents of communication with the MS medical treatment team and symptomatic treatment. Results indicated significant improvements in affective and problem-solving communication, marital satisfaction, and sexual satisfaction after the treatment.

**Table 4:** Treatment effects for SD and psychological measures in intervention studies.

Study	Intervention	Duration	n	SD n (%)	Design	Psychological/SD outcome measures	p
Foley et al. [57]	counselling sessions focusing on sexuality (Education, Symptom Management, sensate focus and cognitive behaviour therapy)	2 month waiting period and 12 weeks intervention phase	9 people with MS / 9 long term partner	nr	quasi-experimental (pre / post calculation	Marital Satisfaction (MAT)	< .001
						Affective Communication (AC)	< .01
						Problem-Solving Communication (PSC)	< .001
						Sexual Satisfaction (SS)	< .05
Blackmore et al. [26]	telephone-administered psychotherapy for depression—cognitive behavioural therapy and supportive emotion focused therapy	16 weeks	127	59 (72.8)	RCT (Secondary analysis of a depression treatment)	UCLA Social Support Inventory(subscale sexual satisfaction)	< .01/ < .001
Khakbazan et al. [27]	PLISSIT contains of sexual counselling based on the Permission, Limited Information, Specific Suggestion, Intensive Therapy	4 weekly sexual counselling sessions (90–120 min per session)	90	100 (100)	RCT	Female sexual function index (FSFI)	< .001

RCT = randomised controlled trial; UCLA = University of California, Los Angeles

In a study which primarily aimed to treat depression in MS, Blackmore et al. [26] investigated the extent of changes in negative and positive partner support to predict sexual satisfaction levels over time in people with MS. The intervention was a 16-week telephone-administered psychotherapy with elements of cognitive behavioural psychotherapy and supportive emotion focused therapy. Results indicated that increased positive partner support was associated with significant improvement in sexual satisfaction over time (β = 0.50, p < .001), as was decreased negative partner support (β = 0.36, p < .01). The authors conclude that both positive and negative partner support have a distinctive role in the outcome of sexual satisfaction for individuals with MS.

Khakbazan et al. [27] evaluated the effectiveness of sexual counselling based on the Permission, Limited Information, Specific Suggestion, Intensive Therapy (PLISSIT) model on the SD of 90 married sexually active women with MS in a randomized clinical trial in Tehran. PLISSIT contents 4 weekly sexual counselling sessions including education, practical hints and exercises, problem solving etc. (90–120 min per session). Results showed improvement in sexual function at 2 and 3 months after the intervention.

### Study quality

Results of the CASP assessments are shown in Tables 5–7.

**Table 5:** CASP quality assessment for case control studies.

	Screening			1	Methods	2	Cases					3	Controls				4	Measures				5	Confounding	6	Results		7	Score
	Did the study address a clearly focused issue?						Were the cases recruited in an acceptable way?						Were the controls selected in an acceptable way?					Was exposure measured to minimise bias?					Have the authors taken account of the potential confounding factors in the design and/or in analysis?		Are results presented transparently?	How precise are the results?		
	The population studied	The risk factors studied	The outcomes considered		Where adequate methods used address the study question?		Are the cases defined precisely?	Were the cases representative of a defined population?	Was there an established reliable system for selecting all the cases?	Was there a sufficient number of cases selected?	Was there a power calculation?		Were the controls representative of defined population	Was the response high? Could non respondents be different in any way?	Are they matched, population based or randomly selected?	Was there a sufficient number of controls selected?		Was the exposure clearly defined and accurately measured?	Do the measurements truly reflect what you want them to (have they been validated)?	Were the measurement methods similar in the cases and controls?	Did the study incorporate blinding where feasible?		Restriction in design, and techniques e.g. modelling, stratified, regression, or sensitivity analysis to correct, control or adjust for confounding factors			Confident intervals? Or other quality calculations		
Ghajarzadeh et al. [39]	Y	Y	Y	**Y**	Y	**Y**	Y	Y	U	Y	N	**Y**	U	U	Y	U	**U**	Y	Y	Y	N	**Y**	N	**N**	Y	Y	**Y**	**5/7**
Gumus et al. [40]	Y	Y	Y	**Y**	Y	**Y**	Y	Y	U	Y	N	**Y**	Y	U	N	U	**U**	Y	Y	Y	N	**Y**	N	**N**	Y	N	**N**	**4/7**
McCabe [45]	Y	Y	Y	**Y**	Y	**Y**	Y	Y	Y	Y	N	**Y**	U	N	Y	U	**U**	Y	Y	Y	N	**Y**	N	**N**	Y	N	**N**	**4/7**
Mc. Cabe et al. [46]	Y	Y	Y	**Y**	Y	**Y**	Y	Y	Y	Y	N	**Y**	U	N	Y	U	**U**	Y	Y	Y	N	**Y**	N	**N**	Y	**N**	**N**	**4/7**

**Table 6:** CASP quality assessment for cohort studies.

	Screening			1	Subjects		2	Detailed questions (a)			3	Detailed questions (b)			4	Confounding		5	Follow up		6	Results	7	Score
	Did the study address a clearly focused issue?				Was the cohort recruited in an acceptable way?			Was exposure measured to minimize bias?				Was outcome measured to minimize bias?							a) Was follow up complete enough?	b) Was the follow up of subjects long enough?		How precise are the results?		
	The population studied	The risk factors studied	The outcomes considered		Was the cohort representative of a defined population?	Was everybody included who should have been included?		Did they use objective measurements?	Do the measurements truly reflect what you want them to (have they been validated)?	Were all the subjects classified into exposure groups using the same procedure		Did they use objective measurements?	Do the measures truly reflect what you want them to (have they been validated)?	Has a reliable system been established for detecting all the cases (for measuring disease occurrence)?		a. Have the authors identified all confounding factors?	b. Have they taken account of the confounding factors in the design and/or analysis?					Confident intervals? Or other quality calculations		
Barak et al. [35]	Y	Y	Y	**Y**	N	U	**N**	N	N	Y	**N**	N	N	N	**N**	N	N	**N**	U	NA	**U**	N	**N**	**1/7**
Dupont, [36]	Y	Y	Y	**Y**	Y	Y	**Y**	Y/N	Y	Y	**Y**	N	Y	Y	**Y**	N	N	**N**	Y	NA	**Y**	N	**N**	5/7
Fragala et al. [37]	Y	Y	Y	**Y**	Y	Y	**Y**	N	Y	Y	**Y**	y	Y	Y	**Y**	Y	Y	**Y**	Y	NA	**Y**	Y	**Y**	7/7
Kisic-Tepavcevic et al. [40]	Y	Y	Y	**Y**	Y	Y	**Y**	N	Y	Y	**Y**	N	Y	Y	**Y**	Y	Y	**Y**	Y	Y	**Y**	Y	**Y**	7/7
Kolzet et al. [41]	Y	Y	Y	**Y**	Y	Y	**Y**	N	Y	Y	**Y**	N	N	Y	**N**	Y	Y	**Y**	Y	NA	**Y**	N	**N**	5/7
Lew-Starowicz et al. [42]	Y	Y	Y	**Y**	Y	N	**N**	N	Y	Y	**Y**	N	Y	Y	**Y**	N	N	**N**	Y	NA	**Y**	Y	**Y**	5/7
McCabe et al. [43]	Y	Y	Y	**Y**	Y	Y	**Y**	N	Y	Y	**Y**	N	Y	Y	**Y**	N	Y	**N**	Y	NA	**Y**	N	**N**	5/7
Mohammadi et al. [46]	Y	Y	Y	**Y**	Y	Y	**Y**	N	Y	Y	**Y**	N	Y	Y	**Y**	N	N	**N**	Y	NA	**Y**	Y	**Y**	6/7
Quaderi et al. [47]	Y	Y	Y	**Y**	Y	Y	**Y**	N	Y	Y	**Y**	N	Y	Y	**Y**	N	N	**N**	Y	NA	**Y**	N	**N**	5/7
Schairer et al. [3]	Y	Y	Y	**Y**	Y	Y	**Y**	N	Y	Y	**Y**	N	Y	Y	**Y**	N	N	**N**	Y	NA	**Y**	N	**N**	5/7
Stepleman et al. [48]	Y	Y	Y	**Y**	Y	Y	**Y**	N	Y	Y	**Y**	N	Y	N	**N**	N	N	**N**	Y	NA	**Y**	N	**N**	4/7
Tepavcevic et al. [49]	Y	Y	Y	**Y**	Y	Y	**Y**	N	Y	Y	**Y**	N	Y	Y	**Y**	N	N	**N**	Y	NA	**Y**	N	**N**	5/7
Vitkova et al. [50]	Y	Y	Y	**Y**	Y	Y	**Y**	N	Y	Y	**Y**	N	Y	Y	**Y**	N	N	**N**	Y	NA	**Y**	Y	**Y**	6/7
Young et al. [51]	Y	Y	Y	**Y**	Y	Y	**Y**	N	Y	Y	**Y**	N	Y	Y	**Y**	N	N	**N**	Y	NA	**Y**	Y	**Y**	6/7
Zivanodiv et al. [52]	Y	Y	Y	**Y**	Y	Y	**Y**	Y/N	Y	Y	**Y**	N	Y	Y	**Y**	N	N	**N**	Y	NA	**Y**	N	**N**	5/7
Zorzon et al. [53]	Y	Y	Y	**Y**	Y	Y	**Y**	N	Y	Y	**Y**	N	Y	N	**N**	N	N	**N**	Y	NA	**Y**	N	**N**	4/7

**Table 7:** CASP quality assessment for intervention studies.

	Scree ning	1	Rando-mization	2	Inclusion		3	Detailed questions			4	Group-characteristics	5	Treat-ment	6	Results		7	Score
	Did the trial address a clearly focused issue?		Was the assignment of patients to treatments randomized?		Were all of the patients who entered the trial properly accounted for at its conclusion?			Were patients, health workers and study personnel ‘blind’ to treatment?				Were the groups similar at the start of the trial?		Aside from the experimental intervention, were the groups treated equally?		Is the primary outcome clearly specified?	How precise are the results?		
			Was the allocation sequence concealed from researchers and patients?		Was the trial NOT stopped early?	Were patients analyzed in the groups to which they were randomised?		Patients?	Health workers?	Study personnel?		Factors that might affect the outcome such as age sex, social class					Confident intervals? Or other quality calculations		
Blackmore et al. [26]	Y	**Y**	Y	**Y**	Y	Y	**Y**	N	Y	Y	**Y**	Y	**Y**	Y	**Y**	N	N	**N**	6/7
Foley et al. [13]	Y	**Y**	N	**N**	Y	NA	**Y**	NA	NA	NA	**NA**	NA	**NA**	NA	**NA**	N	N	**N**	2/7
Khakbazan et al. [27]	Y	**Y**	Y	**Y**	Y	Y	**Y**	N	N	N	**N**	Y	**Y**	U	**U**	N	N	**N**	4/7

Quality ratings for case control studies ranged from 4/7 to 5/7, cohort studies from 1/7 to 7/7 and intervention studies from 2/7 to 6/7.

Heterogeneity in sampling, data collection, and measurement of identified variables were noted between studies.

## Discussion

This is the first review, systematically identifying and summarizing the literature regarding SD in MS in relation to psychological and/or neuropsychological aspects. Apart from mostly cross-sectional studies on associations between SD and these aspects, we also included intervention trials aiming to treat SD and longitudinal studies with respect to psychological and/or neuropsychological aspects. Unfortunately, most studies were small cross-sectional studies limiting the interpretation of results. The paucity of longitudinal and intervention studies further limits the results of this scoping review. It has also to be considered that many cross-sectional studies showing no correlation will not have been published. Therefore, a publication bias towards positive relations cannot be ruled out.

Relations between depression, anxiety, and quality of life and SD in people with MS were commonly researched in the included cross-sectional studies, whereas other psychological factors as cognitive impairment, fatigue, and coping were rarely addressed. There were only three intervention studies carried out for specific SD treatment, of which one was a secondary analysis of a depression treatment study and not targeted on SD in MS. One other was a quasi-experimental designed study with only 9 MS patients and their couples. Only one of the intervention studies regarding SD in MS treatment was a randomized controlled trial by using psychological treatment. Furthermore, there were only two longitudinal studies describing the course of SD in relation to psychological aspects.

Although several studies met our inclusion criteria, it remains difficult to draw clear conclusions from the analysis as studies used many different instruments to measure SD. The most often used instruments to assess SD in MS were the Multiple Sclerosis Intimacy and Sexuality Questionnaire (MSISQ) and the Female sexual function index (FSFI). Both instruments show good psychometric characteristics and are well established in clinical routine and in research [58,59]. The MSISQ is an MS specific instrument whereas the FSFI is generic measures of SD. Studies have to consider the different approaches of questionnaires. As MS specific measures might better account for more MS-specific triggers of SD and may include a couple of modifiable factors moderating SD in MS these tools might be even more sensitive for changes based on interventions. Therefore treatment studies should apply specific tools [27].

Also studies measured and reported a wide range of different psychological aspects (e.g. depression, anxiety, quality of life) using many different instruments. Of the 18 cross-sectional studies nine reported SD in significant relation to depression while two other studies did not find any relations between depression and SD in MS. It has often been reported that depression is the most common psychiatric disorder in people with MS and more prevalent than in other chronic diseases [60]. Many factors might trigger depression in MS such as localization of brain lesions, psychosocial factors, drugs used for treatment, and other disabilities [2]. The relationship between SD in MS and depression could be attributed as part of a vicious cycle where depression contributes to the SD and vice versa which is supported by the fact that SD in MS often occurs in patients in an early stage of disease with no or mild disability [18]. In line with this results also in persons with depression not associated to somatic disease the bidirectional association between depression and SD is reported [61]. In contrast two studies did not find significant relations between depression and SD in MS [37,52]. The study by Dupont [37] investigated SD in 116 MS patients and their partners, but MS patients’ depression scores were far below cut-off scores for depression with only few showing depressive mood. Young et al. [52] also found no significant contribution of depression on SD in MS in 431 MS patients. As a possible explanation, the study used a distinct measure to assess specific aspects of SD (MSISQ-15] and found that depression as the psychological influence of MS impacts worries regarding sexuality, but not directly physical aspects of sexuality.

There is a close relation between depression and anxiety, which is depicted by the fact that five cross-sectional studies observed a relation between SD and anxiety, four of which reported significant positive correlations between SD and anxiety. The impact of anxiety on SD in MS underlines the role of psychological factors on the determination of SD in MS. On the other hand, SD in MS seems also to be characterized and driven by physical impairment as e.g. bladder problems [62] or by specific brain lesion [36]. In addition, SD has been reported to be related to longer MS disease duration and advanced disability [63,64].

In six studies the relation between SD and quality of life (Qol) in MS was investigated and all of them found significant negative relations. In two studies all of the subscales of QoL were calculated in relation to SD total scores and almost all subscales of the QoL measures (e.g. psychological, social and body function) correlated with SD in MS [48,50]. In contrast, Lew-Starowicz et al. [43] focused on the relation between the subscales of SD and QoL showing significant negative correlations in most of the SD subscales (e.g. orgasmic function, erectile function, lubrication, sexual pain) for both, male and female MS patients, but also showing differences between genders. In women a strong association of diminished desire and QoL was detected, but no such correlation was seen in men. A possible explanation could be the more relational determination of female sexuality compared to more function-oriented male sexuality (i.e. relatively less dependent on relationship intimacy and stability). Accessory, we identified more studies with female participants, and male MS patients often are older and corresponding more disabled than women.

Because of the evidence of the massive impact of MS on QoL, the relation between SD and QoL in MS is not remarkable as both physical and psychosocial disease specific factors of SD in MS influence QoL in patients and vice versa.

Four studies looked for relations between SD and fatigue in MS of which only two found significant positive correlations in female and male patients. This is surprising as fatigue is one of the most frequent symptoms reported by people with MS, affecting between 50% and 80% of patients [65]. Due to the small number of studies with inconsistent results, the role of fatigue in relation to SD in MS cannot be interpreted so far, but it is conceivable that presence of fatigue may interfere with sexual life. Very recently, a large international online study with more than 2000 MS patients revealed also independent associations between sexual function and satisfaction and a range of demographic factors, including age, as well as depression risk, antidepressant use, and fatigue in MS patients [66]. In addition, they found associations between SD with modifiable lifestyle factors diet and physical activity.

Two of three studies found significant negative correlations between cognitive performance and SD in MS but only in women with MS [50,53]. Based on the rare investigation of the relation of cognitive impairment on SD in MS no conclusion can be drawn. Apart from this results it should be considered that cognitive distraction in general population is a significant contributor to sexual response problems in both, men and women [9].

Sexuality and couple relationship are strongly associated. All three studies found significant correlations negative between SD and marital or couple relationship. McCabe et al. [44] did not separately looked for gender differences. But both, Lew-Starowicz & Rola [43] and McCabe [45] found more relation between SD and relationship factors in MS women than in MS man. Results are consistent with findings in the general population and Brotto et al. [9] also underlined the interdependence of sexual function between partners, with dysfunction in one partner often contributing to problems in sexual functioning and/or sexual satisfaction for the other. An open question is the importance of good partner relationship for fulfilled sexual life and vice versa.

Sexuality-related communication between people with MS and their MS physicians were investigated by Stepleman et al. [49], who found that more than half of people with MS reported SD, but only a third addressed their sexual concerns to their physician. Findings are in line with results of Lew-Starowicz et al. [67] and have clinical implications considering the high prevalence of SD in people with MS.

Only two longitudinal observations were found, which is surprising considering the progressive nature of the disease and a probable growing impact on both SD and psychological factors [41]. And, there were only three intervention studies focusing with inconclusive outcomes, highlighting the need for more rigorously conducted intervention studies, aiming to support people with MS and SD. In addition, the meaning of sexual activity for MS patients should be more thoroughly studied, through their life cycle and especially in more disabled patients. Not one study addressed these factors.

SD has been described in maJor chronic neurologic conditions as Parkinson disease, stroke and dementia [68–70]. SD dimensions in MS are comparable with dysfunction in Parkinson disease. In Parkinson disease also comparable relations to neuropsychiatric symptoms were found whereas data on SD in dementia are scarce, mainly reporting reduced frequency of sex and erectile dysfunction or SD in relation to hyper sexuality and sexual violation [69]. In stroke a recent study in the USA described that many stroke survivors experienced sexual dysfunction and indicated a desire for additional information and counseling and identified sexuality as an important issue in their post-stroke rehabilitation which is as well neglected in current care [70].

A more general point of view in discussing our findings is on the language used to describe sexuality and SD. How we define sexuality is reflected in the language we use about it, and by screening the literature we found a tendency to define and describe sexuality in relation to the physiological or genital functioning. Psychological factors as “identity”, “role”, “intimacy” which are of course also related to sexuality and SD, but—in most of the studies—are not included. These factors should be more addressed in future work.

We used the CASP tool for quality assessment and overall found satisfactory quality for cohort studies, but revealed lower quality for intervention and case control studies. Major problems were consideration of confounding factors and presentation of study result (especially reporting of confidence intervals).

Our review for the first time systematically reviewed all studies on SD and neuro-/psychological factors in people with MS in a comprehensive scoping review approach. The results may be used to design further association studies which should preferably by longitudinal and use commonly used validated instrument in order to produce reliable and comparable results. We also hope that our results will raise the awareness of clinicians for SD in people with MS and lead to more meaningful clinical encounters. Finally, we expect the results will advertise for future intervention studies targeting SD in people with MS, where obviously a huge potential for non-pharmacological psychological interventions could be identified.

In conclusion, we have shown that neuro-/psychological factors in people with MS and SD is widely recognised and a number of studies have been performed. We found gender differences for both, SD in MS in general and in relation to neuropsychiatric impairment. Differences might be driven by differences in male and female sexuality and also in SD. Gender specific questionnaires according to this differ, those for men are often more functional specific whereas those for women are more related to psychological aspects.

## Supporting information

S1 Table List of used outcome measures for sexual functioning, psychological and neuropsychological assessment in n = 23 studies. (DOCX)

S2 Table Relations between SD and psychological and neuropsychological measures. sm = self made. (DOCX)

S3 Table Prisma checklist. (DOCX)

S1 Search strategy (DOCX)
