# Supplementary material for: Sexual dysfunctions in MS in relation to neuropsychiatric aspects and its psychological treatment: A scoping review
Source: PLoS One. 2018 Feb 27;13(2):e0193381. doi: 10.1371/journal.pone.0193381 (PMC5828449; doi:10.1371/journal.pone.0193381)
Supplement: S1 Table — (DOCX) [file pone.0193381.s001.docx]

**S1 Table: List of used outcome measures for sexual functioning, psychological and neuropsychological assessment in n=23 studies**

| outcome measures | | n |
| --- | --- | --- |
| for sexual functioning | The Multiple Sclerosis Intimacy and Sexuality Questionnaire-19/15 (MSISQ) | 6 |
|  | Female sexual function index (FSFI) | 5 |
|  | selfmade questionnaire | 3 |
|  | Index of sexual satisfaction (ISS) | 2 |
|  | Sexual Satisfaction Survey (SSS) from MS Quality of life Inventory (MSQLI) | 2 |
|  | Marital Satisfaction Inventory (MSI) | 2 |
|  | Sexual Dysfunction Scale SDS) | 1 |
|  | International Index of Erectile Function (IIEF) | 2 |
|  | Sexual Function Scale (SFS) | 1 |
|  | Female Sexual Function Questionnaire (SFQ-28) | 1 |
|  | Sexual Quality of Life Questionnaire—Male and Female versions (SQoL) | 1 |
|  | Szasz sexual functioning scale (SSFS) | 1 |
|  | GolomboFtRust Inventory of Sexual Satisfaction (GRZSS) | 1 |
|  | A specific MS sexuality question (MS-SEX) / wised by the author | 1 |
|  | Sexual Life Importance (VAS 0-10) | 1 |
|  | Incapacity Status Scale (ISS) – subscale sexual function | 1 |
|  | Sexual Disabilities section of the Guy's Neurological Disability Score (GNDS) | 1 |
|  | Nature of the Sexual Problem subscale of the Sexual Dysfunction Scale (SDS) | 1 |
|  | (selfmade) structured interview | 1 |
|  | Common Sexual Activity | 1 |
|  | University of California Social Support Inventory (UCLA) (subscale sexual satisfaction) | 1 |
| neuro-psychological assessment | Mini Mental State Examination (MMSE) | 2 |
|  | Screening Examination for Cognitive Impairment in Multiple Sclerosis (SECIMS) | 1 |
| depression and anxiety | Beck Depression Inventory (BDI) | 7 |
|  | Hamilton Anxiety Rating Scale (HARS) | 4 |
|  | Hamilton Depression Rating Scale (HDRS) | 3 |
|  | HospitaI Anxiety and Depression Scale (HADS) | 2 |
| quality of life | Multiple Sclerosis Quality of Life-54 (MSQOL-54) | 2 |
|  | Short Form-12 (SF-12) | 2 |
|  | Short Form Health Survey (SF-36) | 1 |
|  | World Health Organization Quality of Life-100 Scale (WHOQOL-100) | 1 |
| marital, relationship and social support | Marital Satisfaction Inventory (MSI) | 2 |
|  | selfmade relationship questionnaire | 1 |
|  | UCLA Social Support Scale | 1 |
|  | Golombok-Rust Inventory of Marital State (GRIMS) | 1 |
|  | Female Sexual Function Questionnaire (SFQ-28) | 1 |
| coping | Ways of Coping Questionnaire (WOCQ) | 2 |
|  | Acceptance of Illness Scale (AIS) | 1 |
| fatigue | Fatigue Severity Scale (FSS) | 2 |
|  | Selfmade structural interview | 1 |
|  | Neurological Fatigue Index-MS (NFI-MS) | 1 |
| others | Acceptance of lllness Scale (AZS) | 1 |
|  | Body image related SD (item 7 of MSISQ) | 1 |
|  | semistructural psychiatric interview | 1 |
|  | Mental Health Inventory (MHI) | 1 |
|  | Patient Satisfaction Questionnaire (PSQ-18) | 1 |
|  | Perceived Self-Efficacy in Patient-Physician Interactions—Sex (PEPPI-S) | 1 |
