# Supplementary material for: Sexual dysfunctions in MS in relation to neuropsychiatric aspects and its psychological treatment: A scoping review
Source: PLoS One. 2018 Feb 27;13(2):e0193381. doi: 10.1371/journal.pone.0193381 (PMC5828449; doi:10.1371/journal.pone.0193381)
Supplement: S2 Table — (DOCX) [file pone.0193381.s002.docx]

**S2 Table: Relations between SD and psychological and neuropsychological measures**

| **Calculated outcomes in MS regarding SD and psychology in MS** | **p-value** | **OR (CI), r, R** |
| --- | --- | --- |
| ***relation between Sexual dysfunction and depression*** |  |  |
| **Mohammadi et al. 2013** |  |  |
| BDI * FSFI (total) | <.001 | OR (CI): 1.11 (1.07-1.15) |
| BDI * FSFI (orgasmic dysfunction) | 0.023 | OR (CI): 1.03 (1.00-1.06) |
| BDI * FSFI (sexual satisfaction) | 0.012 | OR (CI): 1.04 (1.00-1.07) |
| **Zorzon et al. 2003** |  |  |
| HDRS * sm structured interview | <.0001 | R: .41 |
| **Zivadinov et al. 1999** |  |  |
| HDRS * sm questionnaire (SD) (female) | .0018 | r: .40 |
| **Lew-Starowicz et al. 2014** |  |  |
| BDI * IIEF (total score) (male) | .0167 | r: -.46 |
| BDI * IIEF (desire) (male) | .0038 | r: -.36 |
| BDI * IIEF (erectile function) (male) | .0252 | r: -.40 |
| BDI * IIEF (overall satisfaction) (male) | .0092 | r: -.42 |
| BDI * SFQ (total score) (female) | .0101 | r: -.29 |
| BDI * SFQ (desire) (female) | .0205 | r: -.20 |
| BDI * SFQ (sensation) (female) | .0416 | r: .-22 |
| BDI * SFQ (cognitive arousal) (female) | .0223 | r: -.24 |
| BDI * SFQ (orgasm) (female) | .0010 | r: -.34 |
| BDI * SFQ (enjoyment) (female) | .0031 | r: -.31 |
| BDI * SFQ (partner domain) (female) | .0091 | r: -.23 |
| **Tepavcevic et al. 2008** |  |  |
| HDRS * SSFS (male) | <.01 | r: .58 |
| HDRS * SSFS (female) | <.01 | r: .39 |
| **Ghajarzadeh et al. 2013** |  |  |
| BDI * FSFI (desire) | <.001 | r: -.46 |
| BDI * FSFI (arousal) | <.001 | r: -.46 |
| BDI * FSFI (lubrication) | <.001 | r: -.27 |
| BDI * FSFI (orgasm) | <.001 | r: -.32 |
| BDI * FSFI (satisfaction) | <.001 | r: -.32 |
| BDI * FSFI (pain) | <.001 | r: -.35 |
| BDI * FSFI (total score) | <.001 | r: -.42 |
| BDI * FSFI (totalscore) | .04 | OR (CI): 0.93 (0.88 - 0.99) |
| **Barak et al. 1996** |  |  |
| BDI * sm questionnaire (SD) | .001 | r: .68 |
| **Fragala et al. 2014** |  |  |
| HDRS * IIEF (erectile function) (male) | <.05 | OR (CI): −0.24 (−0.554 - −0.01) |
| HDRS * IIEF (intercourse satisfaction) (male) | <.05 | OR (CI): −0.25 (−0.26 - −0.01) |
| HDRS * FSFI (desire) (female) | <.01 | OR (CI): −0.63 (−0.18 - −0.10) |
| HDRS * FSFI (arousal) (female) | <.01 | OR (CI): −0.61 (−0.21 - −0.11) |
| HDRS * FSFI (lubrication) (female) | <.01 | OR (CI): −0.54 (−0.20 - −0.09) |
| HDRS * FSFI (orgasm) (female) | <.01 | OR (CI): −0.54 (−0.15 - −0.71) |
| HDRS * FSFI (satisfaction) (female) | <.01 | OR (CI): −0.63 (−0.21 - −0.12) |
| HDRS * FSFI (pain) (female) | <.01 | OR (CI): −0.54 (−0.25 - −0.12) |
| HDRS * FSFI (total score) (female) | <.01 | OR (CI): −0.62 (−1.18 - −0.62) |
| HDRS * MSISQ Primary Sexual Dysfunction | <.01 | OR (CI): 0.43 (0.22 - 0.50) |
| HDRS * MSISQ Secondary Sexual Dysfunction | <.01 | OR (CI): 0.48 (0.33 - 0.62) |
| HDRS * MSISQ Tertiary Sexual Dysfunction | <.01 | OR (CI): 0.30 (0.11 - 0.39) |
| **Gumus et al. 2014** |  |  |
| BDI * FSFI (total score) | >.001 | r: −0.884 |
| ***relation between Sexual dysfunction and anxiety*** | | |
| **Zorzon et al. 2003** |  |  |
| HARS * sm structured interview | .002 | R: .38 |
| **Zivadinov et al. 1999** |  |  |
| HARS * sm questionnaire (SD) (female) | .0017 | r: .40 |
| **Tepavcevic et al. 2008** |  |  |
| HARS * SSFS (male) | <.01 | r: .51 |
| HARS * SSFS (female) | <.01 | r: .40 |
| **Fragala et al. 2014** |  |  |
| HARS * FSFI (desire) (female) | <.01 | OR (CI): −0.63 (−0.29 -−0.10) |
| HARS * FSFI (arousal) (female) | <.01 | OR (CI): −0.63 (−0.22 - −0.12) |
| HARS * FSFI (lubrication) (female) | <.01 | OR (CI): −0.58 (−0.22 - −0.10) |
| HARS * FSFI (orgasm) (female) | <.01 | OR (CI): −0.57 (−0.16 - −0.08) |
| HARS * FSFI (satisfaction) (female) | <.01 | OR (CI): −0.65 (−0.230 - −0.13) |
| HARS * FSFI (pain) (female) | <.01 | OR (CI): −0.56 (−0.26 - −0.13) |
| HARS * FSFI (total score) (female) | <.01 | OR (CI): −0.62 (−1.37 - −0.61) |
| HARS * MSISQ Primary Sexual Dysfunction | <.01 | OR (CI): 0.35 (0.14 - 0.40) |
| HARS * MSISQ Secondary Sexual Dysfunction | <.01 | OR (CI): 0.37 (0.18 - 0.47) |
| HARS * MSISQ Tertiary Sexual Dysfunction | <.01 | OR (CI): 0.26 (0.07 - 0.31) |
| ***relation between SD and coping*** | |  |
| **McCabe 2002** |  |  |
| WOCQ (focusing on the positive) * ISS (male) | <.05 | beta: -.34 |
| WOCQ (focusing on the positive) * SDS (male) | <.05 | beta: -.26 |
| WOCO (detachement) * ISS (female) | <.05 | beta: .22 |
| WOCO (cognitive functioning) * ISS (female) | <.01 | beta: -.28 |
| WOCQ (focusing on the positive) * SDS (female) | <.05 | beta: -.24 |
| WOCQ (problem focused coping) * SDS (female) | <.05 | beta: .32 |
| WOCO (cognitive functioning) * SDS (female) | <.01 | beta: -.18 |
| ***relation between sexual dysfunction and fatigue*** | | |
| **Zivadinov et al. 1999** |  |  |
| sm interview * sm questionnaire (SD) (female) | .0284 | r: .30 |
| **Tepavcevic et al. 2008** |  |  |
| FSS * SSFS (male) | <.05 | r: .44 |
| FSS * SSFS (female) | <.01 | r: .70 |
| ***relation between sexual dysfunction and cognitive performance*** |  |  |
| **Zivadinov et al. 1999** |  |  |
| MMSE * sm questionnaire (SD) (female) | .0280 | r: .30 |
| **Tepavcevic et al. 2008** |  |  |
| MMSE * SSFS (female) | <.01 | r: -.39 |
| ***relation between sexual dysfunction and quality of life*** |  |  |
| **Lew-Starowicz et al. 2014** |  |  |
| SQoL (male) * IIEF (erectile function) | .0039 | r: .48 |
| SQoL (male) * IIEF (orgasmic function) | .0364 | r: .34 |
| SQoL (male) * IIEF (intercourse satisfaction) | .0162 | r: .42 |
| SQoL (male) * IIEF (overall satisfaction) | .0303 | r: .34 |
| SQoL (male) * IIEF (sex function total) | .0002 | r: .63 |
| SQoL (female) * SFQ-28 (desire) | <.0001 | r: .56 |
| SQoL (female) * SFQ-28 (arousal sensation) | <.0001 | r: .47 |
| SQoL (female) * SFQ-28 (lubrication) | <.0001 | r: .48 |
| SQoL (female) * SFQ-28 (cognitive arousal) | <.0001 | r: .48 |
| SQoL (female) * SFQ-28 (orgasmic function) | <.0001 | r: .55 |
| SQoL (female) * SFQ-28 (sexual pain) | <.0001 | r: .48 |
| SQoL (female) * SFQ-28 (overall satisfaction) | <.0001 | r: .64 |
| SQoL (female) * SFQ-28 (partner domain) | <.0001 | r: .41 |
| SQoL (female) * SFQ-28 (sex function total) | <.0001 | r: .69 |
| **Tepavcevic et al. 2008** |  |  |
| MSQLI (physical health) * SSFS (male) | <.05 | r: -.59 |
| MSQLI (physical role limitations) * SSFS (male) | <.05 | r: -.54 |
| MSQLI (social function) * SSFS (male) | <.05 | r: -.55 |
| MSQLI (health distress) * SSFS (male) | <.05 | r: -.55 |
| MSQLI (sexual function) * SSFS (male) | <.05 | r: -.91 |
| MSQLI (social function satisfaction) * SSFS (male) | <.05 | r: -.86 |
| MSQLI (physical role limitations) * SSFS (female) | <.05 | r: -.32 |
| MSQLI (emotional role limitations) * SSFS (female) | <.05 | r: -.29 |
| MSQLI (emotional well being) * SSFS (female) | <.05 | r: -.58 |
| MSQLI (energy) * SSFS (female) | <.05 | r: -.60 |
| MSQLI (physical health) * SSFS (female) | <.05 | r: -.64 |
| MSQLI (Health perception) * SSFS (female) | <.05 | r: -.46 |
| MSQLI (social function) * SSFS (female) | <.05 | r: -.58 |
| MSQLI (health distress) * SSFS (female) | <.05 | r: -.56 |
| MSQLI (cognitive function) * SSFS (female) | <.01 | r: -.22 |
| MSQLI (sexual function) * SSFS (female) | <.05 | r: -.71 |
| MSQLI (Change in health) * SSFS (female) | <.05 | r: -.37 |
| MSQLI (social function satisfaction) * SSFS (female) | <.05 | r: -.62 |
| MSQLI (total score) * SSFS (female) | <.05 | r: -.67 |
| **Schairer et al. 2014** |  |  |
| SF-12 (PCS - physical components summary)* MSISQ | <.01 | r: -.46 |
| SF-12 (MCS - mental components summary)* MSISQ | <.01 | r: -.37 |
| **Quaderi et al. 2013** |  |  |
| MSQLI (physical role limitations) * MSISQ | <.01 | r: -.44 |
| MSQLI (emotional role limitations) * MSISQ | <.01 | r: -.32 |
| MSQLI (emotional well being) * MSISQ | <.01 | r: -.28 |
| MSQLI (energy) * MSISQ | <.01 | r: -.42 |
| MSQLI (physical health) * MSISQ | <.01 | r: -.49 |
| MSQLI (Health perception) * MSISQ | <.01 | r: -.43 |
| MSQLI (social function) * MSISQ | <.01 | r: -.54 |
| MSQLI (health distress) * MSISQ | <.01 | r: -.26 |
| MSQLI (cognitive function) * MSISQ | <.01 | r: -.41 |
| MSQLI (sexual function) * MSISQ | <.01 | r: -.78 |
| MSQLI (Change in health) * MSISQ | <.01 | r: -.32 |
| MSQLI (social function satisfaction) * MSISQ | <.01 | r: -.57 |
| MSQLI (pain) * MSISQ | <.01 | r: -.40 |
| MSQLI (total score) * MSISQ | <.01 | r: -.42 |
| MSQLI (physical health composit)* MSISQ | <.01 | r: -.64 |
| MSQLI (physical health composit)* MSISQ | <.01 | r: -.43 |
| **Vitkova et al. 2014** |  |  |
| SF-36 (PCS - physical components summary)* ISS | .004 | r: -.27 |
| SF-36 (MCS - mental components summary)* ISS | .002 | r: -.30 |
| **Kolzet et al. 2015** |  |  |
| SF-12 (PCS - mental components summary)* MSISQ (item 7) | <.01 | r: -.367 |
| ***relation between sexual dysfunction and relationship*** |  |  |
| **McCabe et al. 1996** |  |  |
| sm (feel about relationship) * sm (partner concern about sex) | <.001 | r: -.39 |
| sm (feel about relationship) * sm (frequency of intercourse) | <.001 | r: .45 |
| sm (feel about relationship) * sm (frequencies of intercourse last year) | <.001 | r: .48 |
| **McCabe 2002** |  |  |
| KMS (relationship satisfaction) * ISS (sexual satisfaction) (male) | <.001 | r: .72 |
| KMS (relationship satisfaction) * SDS (sexual difficulties) (female) | <.01 | r: -.22 |
