# Supplementary material for: Sexual dysfunctions in MS in relation to neuropsychiatric aspects and its psychological treatment: A scoping review
Source: PLoS One. 2018 Feb 27;13(2):e0193381. doi: 10.1371/journal.pone.0193381 (PMC5828449; doi:10.1371/journal.pone.0193381)
Supplement: S1 Search strategy — (DOCX) [file pone.0193381.s004.docx]

**S1 Search strategy**

The search strategy for MEDLINE using DBIS was as follows:

1. “multiple sclerosis.mp. or exp Multiple Sclerosis/”

2. “sexual function disturbances.mp.”

3.” sexual disorders.mp.”

4. “sexual symptoms.mp.”

5. “lubr*.mp.” 6.” orgasm.mp.”

7. “sexual satisfaction.mp.”

8. “sexu*.mp.”

9. “2 or 3 or 4 or 5 or 6 or 7 or 8”

10. “1 and 9”

11. “limit 10 to yr="1985 –Current””.
